# Associations of Clinical and Molecular Characteristics with the Response to Immune Checkpoint Blockade in Advanced Gastric Cancers

**DOI:** 10.1155/2022/2162229

**Published:** 2022-03-04

**Authors:** Xiaoqing Xu, Jingjing Li, Yiding Gao, Wangxia Lv, Qing Wei, Xiaowan Tang, Jinlin Hu, Xing Yuan, Wei Wu, Lingnan Zhang, Cong Luo, Lei Chen, Jieer Ying, Xiu Zhu, Qi Xu

**Affiliations:** ^1^Department of Medical Oncology, The Second Clinical Medical College of Zhejiang Chinese Medical University, Hangzhou, China; ^2^Department of Hepato-Pancreato-Biliary & Gastric Medical Oncology, Cancer Hospital of the University of Chinese Academy of Sciences (Zhejiang Cancer Hospital), Institute of Basic Medicine and Cancer (IBMC), Chinese Academy of Sciences, Hangzhou, Zhejiang, China; ^3^Department of Oncology, The Second Affiliated Hospital of Jiaxing University, Jiaxing, China; ^4^Department of Colorectal Medical Oncology, Cancer Hospital of the University of Chinese Academy of Sciences (Zhejiang Cancer Hospital), Institute of Basic Medicine and Cancer (IBMC), Chinese Academy of Sciences, Hangzhou, Zhejiang, China; ^5^Department of Hematological Oncology, Taizhou First People's Hospital, Taizhou, China; ^6^Department of Pathology, Cancer Hospital of the University of Chinese Academy of Sciences (Zhejiang Cancer Hospital), Institute of Basic Medicine and Cancer (IBMC), Chinese Academy of Sciences, Hangzhou, Zhejiang, China; ^7^Department of Medical Oncology, Zhejiang Medical & Health Group Hangzhou Hospital, Hangzhou, China; ^8^Radiology Department, Cancer Hospital of the University of Chinese Academy of Sciences (Zhejiang Cancer Hospital), Institute of Basic Medicine and Cancer (IBMC), Chinese Academy of Sciences, Hangzhou, Zhejiang, China

## Abstract

**Purpose:**

Immunotherapy provides a new treatment option for advanced gastric cancer (AGC). This study aims to explore the response markers of immunotherapy in AGCs.

**Methods:**

Next-generation sequencing was performed on 44 AGC patients who received immune checkpoint inhibitors and the associations between their outcomes after combination immunotherapy, and the clinicopathological/molecular characteristics were analyzed.

**Results:**

The current study cohort had a median progression-free survival (PFS) of 5.9 months, an overall survival (OS) of 12.1 months, and an objective response rate (ORR) of 36.4%. Through multivariable analysis of the clinical characteristics, primary tumor resection (HR = 2.66, 95% CI: 1.06–6.70, *p*=0.037) and increased proportion of lymphocytes after combination immunotherapy (HR = 0.40, 95% CI: 0.16–0.99, *p*=0.048) were revealed as independent predictors for patient outcomes. All the 18 patients who underwent genetic profiling were microsatellite-stable with a median TMB of four mutations per Mb. ATM alterations, PI3K pathway mutations, increased TMB, and positive PD-L1 expression were associated with the increased trend of PFS and ORR. According to the combination of baseline lymphocyte count, ATM mutation, TMB status, and PD-L1 expression, patients were stratified into higher- and lower-risk groups, with the lower-risk group showing improved PFS (HR = 4.7*e*−10, 95% CI: 0–inf, *p*=0.02) and ORR (75% vs. 0%, *p*=0.007).

**Conclusion:**

Several highly relevant potential biomarkers predictive of immunotherapy response in AGC patients have been identified in this research.

## 1. Introduction

Advanced gastric cancers (AGCs) are characterized by poor prognosis and lack of effective treatments. The 5-year survival rate of the AGC patients is 5–20%, with the median overall survival (OS) being less than one year [1–3]. Although chemotherapy is the most widely used treatment option for patients with advanced or metastatic gastric cancers (GCs), there is still no single standard of care established for such patients. Several targeted therapeutic agents have been developed for GCs. The overall survival of patients with HER2-positive AGCs can be improved by trastuzumab in combination with chemotherapy compared with chemotherapy alone [4]. Ramucirumab (an anti-VEGFR-2 antibody) has shown survival benefits either as a single agent or in combination with chemotherapy in patients who progressed after first-line chemotherapy [5, 6]. With the advent of immunotherapy, AGC patients now have more treatment options. At present, the use of immune checkpoint inhibitors alone has been proven to be effective in patients with heavily pretreated AGCs [7–9]. Phase III trials have also shown promising results, supporting the safety and efficacy of immunotherapy in combination with chemotherapy in the first-line setting [10–14]. However, similar to other types of cancers, there are still a majority of unselected patients nonresponsive to immunotherapy [7, 8]. Combination therapies can improve the therapeutic efficacy, and finding biomarkers for immunotherapy across diverse cancers has always been an area of extensive research. Multiple biomarkers predictive of the response to pembrolizumab monotherapy have emerged, including programmed cell death-ligand 1 (PD-L1) expression [7, 8] and microsatellite instability-high (MSI) or deficient mismatch repair (dMMR) [15, 16]. However, PD-L1 expression is associated with conflicting results as a predictive biomarker in AGC patients [7, 17, 18]. Therefore, additional biomarkers are particularly needed to refine the stratification of patients and improve the overall clinical outcomes.

In this study, we evaluated the clinical responses of a cohort of AGC patients who received immunotherapy combined with other anticancer agents. We also performed association studies on their clinicopathological characteristics as well as genetic and immune-related features in an attempt to identify potential biomarkers associated with the response of combination therapy.

## 2. Materials and Methods

### 2.1. Study Design and Participants

A single-center study was conducted at Zhejiang Cancer Hospital under a human research ethics committee-approved protocol. 44 AGC patients who received anti-PD-1 therapy combined with either chemotherapy or antiangiogenic agents from April 2018 to February 2020 were included. Written informed consent was obtained from each patient in accordance with relevant institutional regulations. The clinicopathological information of each patient was retrospectively reviewed.

### 2.2. Next-Generation Sequencing

Next-generation sequencing assays were conducted as described previously [19]. In brief, DNA was extracted from FFPE sections and quantified by a PicoGreen fluorescence assay (Invitrogen). DNA fragmentation was performed by sonication, followed by purification using the AMPure XP Beads (Agencourt). Sequencing libraries were constructed with the NEBNext kits (NEB), amplified with HiFi (Kapa), quantified by qPCR (Kapa SYBR Fast), and sized on a LabChip GX (Caliper). Hybridization was done using a pool of 23,685 individually synthesized oligonucleotides (Integrated DNA Technology), which targeted exons of 287 cancer genes and introns of 19 fusion genes. The captured library was amplified, purified, quantified by qPCR (Kapa), and sized on a LabChip GX (Caliper). Normalized libraries were pooled and sequenced on the Illumina HiSeq 2000 instrument.

### 2.3. Mutation Detection

Sequence data were aligned to the human genome (hg19) using a BWA aligner [20]. PCR deduplication was performed using Picard. Local alignment optimization was conducted using GATK [21]. For base substitutions, a Bayesian method for detection was used and the reads with mapping quality <25 or base calling quality ≤2 were discarded. After filtering out the strand bias, variants were called at mutant allele frequencies (MAF) ≥5% (or ≥1% at hotspots). For indels, the de Brujin approach was used for de novo local assembly in each targeted exon. After strand bias filtering, indels were called with MAF ≥3% at hotspots. The copy number alterations were detected based on the log-ratio profile of each sample, with amplifications called at ≥6 copies (7 for triploid; 8 for tetraploid tumors) and homozygous deletions called at zero copies. Fusion events were detected through analysis of the chimeric read pairs, followed by filtering for mapping quality >30, distributing alignment positions and performing functional annotations.

### 2.4. Statistical Analysis

Quantitative data were presented as median (range) or number of patients (percentage). Fisher's exact test was used to compare the proportions between groups. Survival analysis was performed using Kaplan–Meier curves, *p* value was determined with the log-rank test, and hazard ratios (HRs) were calculated by the Cox proportional hazards model. A two-sided *p* value of less than 0.05 was considered significant for all tests unless indicated otherwise. Univariable analysis was performed to find out the associations between different variables and PFS, and results were presented as HRs and their 95% confidence intervals (CIs). All analyses were performed with *R* 3.4.0.

## 3. Results

### 3.1. Patient Characteristics

A total of 44 AGC patients receiving anti-PD-1 therapy were included in the study cohort, and their baseline clinical characteristics were summarized and presented in Table 1 (with the median age being 60 years, ranging from 30 to 74; 66% male patients). The liver was found as the most common site of metastasis besides lymph nodes in 56.8% of patients. Among patients with known differentiation status (*n* = 35), 15 (42.9%) had moderately differentiated tumors and 20 (57.1%) had poorly differentiated tumors. 23 (patients (52.3%) received no prior therapies and 21 patients (47.7%) had received at least one systemic therapy. All patients in the study cohort received combination therapy, with 54.5% of them receiving anti-PD-1 therapy combined with antiangiogenic agents and 45.5% of them being treated with chemotherapy.

### 3.2. Efficacy of the Combination Therapy

**Figure 1:**
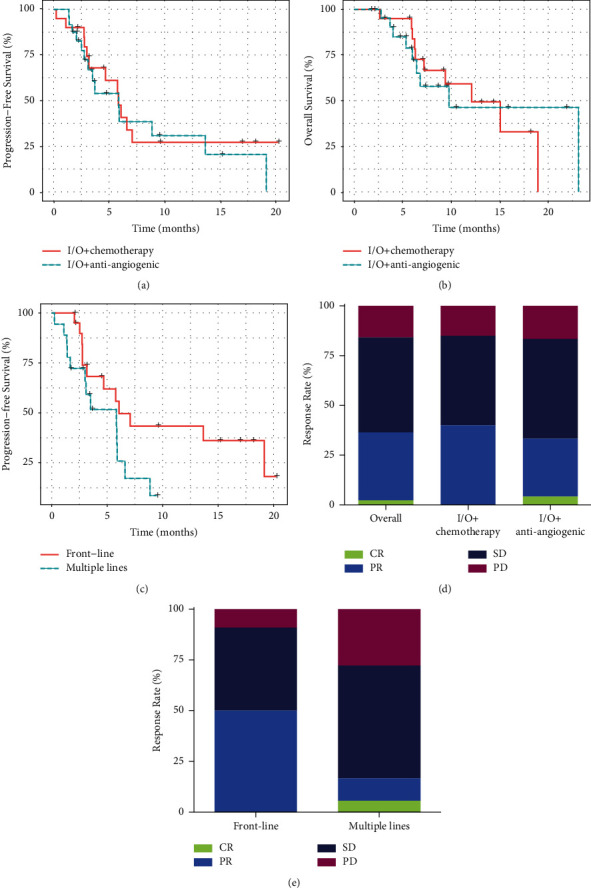
Efficacy of combination immunotherapy in AGC patients. (a, b) Kaplan–Meier plots of (a) progression-free survival and (b) overall survival for patients treated with immunotherapy combined with different antitumor agents. (c) Kaplan–Meier plot of progression-free survival for patients treated with combination immunotherapy in the front-line or subsequent-line settings. (d) Response rates for patients treated with immunotherapy combined with different antitumor agents. CR, complete response; PR, partial response; SD, stable disease; PD, progressive disease. (e) Response rates for patients treated with combination immunotherapy in the front-line or subsequent-line settings.

At the time of data cutoff, 26 patients (59.1%) had progressed and 19 (43.2%) had died. The median progression-free survival (PFS) and overall survival (OS) were 5.9 months and 12.1 months, respectively (Figures S1(a) and S1(b)). There was no significant difference in PFS (*p*=0.64) or OS (*p*=0.95) among patients receiving different types of combination therapies (Figures 1(a) and 1(b)). An increase in PFS was displayed in patients who received combination immunotherapy in the first-line setting compared to those who received prior lines of therapies (mPFS = 7.1 versus 5.8 months, HR = 0.45, 95% CI = 0.20–1.03, *p*=0.053) (Figure 1(c)), but no clear difference in OS was observed (HR = 0.64, 95% CI = 0.25–1.67, *p*=0.36) (Figure S1(c)).

Overall, the objective response rate (ORR) was 36.4% and the disease control rate (DCR) was 84.1%. There was only one (2.3%) patient with complete response (CR), 15 (34.1%) with partial response (PR), and 21 (47.7%) with stable disease (SD) (Figure 1(d)). The DCR of all treatment regimens were quite consistent, ranging from 83.4% to 85% (Figure 1(d)). The ORR of patients who received front-line immunotherapy was also higher than those who had progressed on two or more lines of systemic therapies (ORR, 48% vs. 24%, Fisher's exact test *p*=0.13) (Figure 1(e)). Among the 23 patients in the first-line setting, 65.2% (15/23) received immunotherapy combined with chemotherapy, and 34.8% (8/23) received immunotherapy combined with antiangiogenic agents.

### 3.3. Associations between Clinical Features and Immunotherapy Outcomes

**Figure 2:**
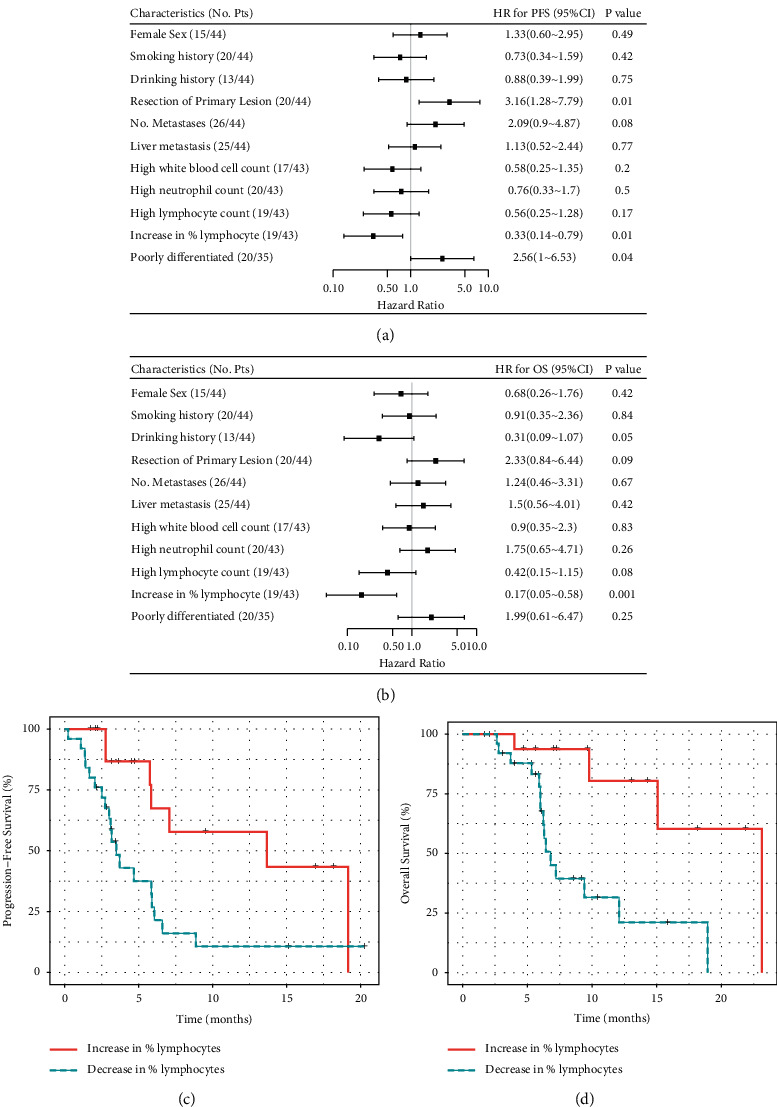
Clinical characteristics associated with immunotherapy responses. (a, b) Forest plots of hazard ratios for (a) progression-free survival and (b) overall survival showing subgroups with the indicated clinical characteristics. (c, d) Kaplan–Meier plots of (c) progression-free survival and (d) overall survival for changes in the lymphocyte proportion in the blood.

Next, we analyzed the correlations between clinicopathologic features and immunotherapy outcomes. Features including sex, smoking history, or metastasis events were not significantly correlated with PFS or OS (Figures 2(a), 2(b)). Patients who had experienced primary lesion resection had poorer PFS (HR = 3.16, 95% CI: 1.28–7.79, *p*=0.01, Figure 2(a) and.  S2(a)) and a trend of worse OS (HR = 2.33, 95% CI: 0.84–6.44, *p*=0.09, Figure 2(b) and.  S2(b)) than those who had not. Among patients with evaluable differentiation status, patients with poorly differentiated tumors showed poorer PFS (HR = 2.56, 95% CI: 1–6.53, *p*=0.04, Figures 2(a) and.  S2(c)) than those with moderately differentiated tumors, but no significant difference in OS (Figure 2(b)) was seen in these patients. We also assayed for various immune cell counts at baseline and after immunotherapy. At baseline, higher levels of total white blood cell counts (≥6000 cells/*μ*L; HR = 0.58, 95% CI: 0.25–1.35, *p*=0.2) and neutrophils (≥3500 cells/*μ*L; HR = 0.76, 95% CI: 0.33–1.7, *p*=0.5) as well as lymphocytes (≥1500 cells/*μ*L; HR = 0.56, 95% CI: 0.25–1.28, *p*=0.17) were all associated with the trends of longer PFS (Figure 2(a) and.  S2(d)). The lymphocyte count at baseline was also associated with increased OS (HR = 0.42, 95% CI: 0.15–1.15, *p*=0.08, Figure 2(b) and.  S2(e)). We noted that patients with increased lymphocyte proportion showed more significant PFS (HR = 0.33, 95% CI: 0.14–0.79, *p*=0.04, Figures 2(a) and 2(c)) and OS improvements (HR = 0.17, 95% CI: 0.05–0.58, *p*=0.001, Figures 2(b) and 2(d)) than those with decreased lymphocyte proportion. In addition, primary lesion resection (20% vs. 50%, Fisher's exact test *p*=0.06), poorly differentiated tumors (15% vs. 67%, *p*=0.004), and decreased proportion of lymphocytes (20% vs. 58%, *p*=0.01) were all associated with the decrease of ORRs in patients. Multivariable Cox analysis indicated that primary lesion resection (HR = 2.66, 95% CI: 1.06–6.70, *p*=0.037) and the altered lymphocyte proportion (HR = 0.40, 95% CI: 0.16–0.99, *p*=0.048) were all independent predictors for PFS.

### 3.4. Associations between Molecular Features and Immunotherapy Outcomes

**Figure 3:**
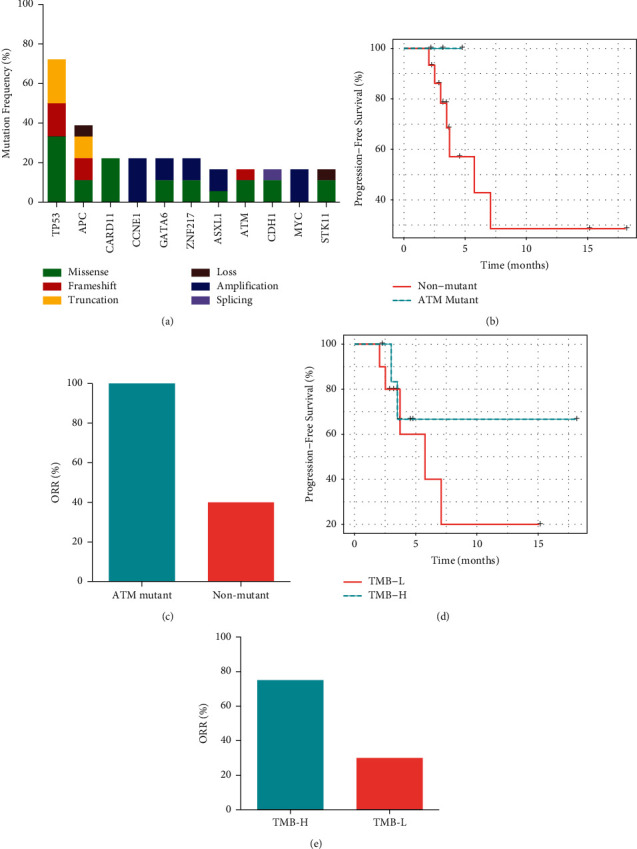
Molecular characteristics associated with immunotherapy responses. (a) The most frequently mutated genes in the AGC cohort. (b) Kaplan–Meier plot of progression-free survival for patients with ATM mutations. (c) Response rates for patients with ATM mutations. (d) Kaplan–Meier plot of progression-free survival for patients with different TMB status. (e) Response rates for patients with different TMB status.

A total of 18 patients were genetically profiled, and their clinical characteristics were similar to those of the entire cohort (Table 1). All patients had microsatellite-stable cancers and relatively low TMB (median TMB = 4 mutations/Mb). Consistent with other studies [22, 23], TP53, APC, CCNE1, ATM, and CDH1 were among the most highly altered genes in our cohort (Figure 3(a)). An increased trend of PFS was shown in patients with ATM, CCNE1, or CDH1 alterations (Figures 3(b) and S3(a) and S3(b)), while a decreased trend of PFS was shown in patients with APC and MYC alterations (Figures S3(c) and S3(d)). In addition, partial responses were observed in all the three ATM-mutated patients (ORR = 100% vs. 40%, Fisher's exact test *p*=0.2, Figure 3(c)). Pathway analysis revealed that the deregulation of the PI3K pathway was associated with a better PFS outcome (HR = 0.32, 95% CI: 0.06–1.66, *p*=0.15, Figure S3(e)).

Next, we evaluated several known immunotherapy response-related biomarkers in our cohort. Although the overall TMB was relatively low, patients with higher TMB (≥5 mutations/Mb) had a trend of longer PFS (HR = 0.56, 95% CI: 0.11–2.98, *p*=0.50, Figure 3(d)) and increased ORR (75% vs. 30%, Fisher's exact test *p*=0.15, Figure 3(e)) when they were subdivided based on the TMB results. Immunohistochemical data for PD-L1 expression were available from 15 patients, of which 5 were identified with PD-L1-positive tumors (combined positive score [CPS] ≥1). Patients with positive PD-L1 expression also had an increased trend in PFS (HR = 9.8*e*−10, 95% CI: 0–Inf, *p*=0.15, Figure S4(a)) and ORR (60% vs. 30%, Fisher's exact test *p*=0.35, Figure S4(b)).

### 3.5. Combinatorial Features in Predicting the Responses to Immunotherapy

**Figure 4:**
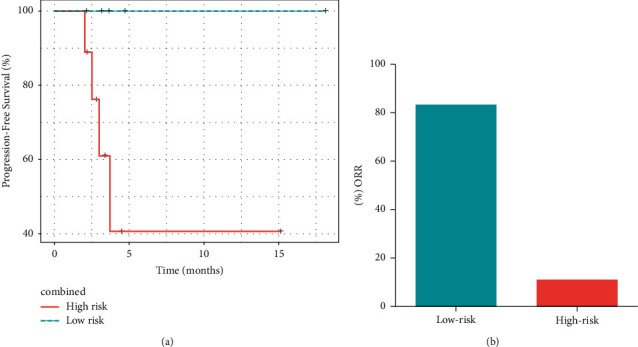
Integration of clinical and molecular characteristics in predicting the immunotherapy responses. (a) Two risk groups were identified with a total of four characteristics (baseline lymphocyte count, TMB status, PD-L1 expression status, and ATM mutation status) factored in. Kaplan–Meier plot of progression-free survival for patients in different risk groups. (b) Response rates for patients in different risk groups.

Finally, we tested the combinations of relevant clinical and molecular features in order to better predict the immunotherapy outcomes. Integrated analysis of baseline lymphocyte count, ATM alterations, TMB status, and PD-L1 expression led to a more refined stratification of patients benefitting from immunotherapy. Each tumor was scored based on these four features, with one point given when a positive predictor appeared. Patients were subdivided into two groups, with the high-risk group scored 0-1 and the low-risk group scored 2-3. Compared with the high-risk group, the PFS improvement was more prominent in the low-risk group (HR = 7.85*e*−10, 95% CI: 0–Inf, *p*=0.02, Figure 4(a)). In addition, the low-risk group was enriched with patients who responded to immunotherapy (83% vs. 11%, Fisher's exact test *p*=0.01, Figure 4(b)).

## 4. Discussion

Immunotherapy, particularly the use of immune checkpoint inhibitors, has emerged as a new and promising therapeutic option for patients with AGCs. Here, we evaluated the efficacy of combination immunotherapy for AGC patients and explored the clinical and molecular characteristics associated with their immunotherapy responses. In our AGC cohort, compared with any single risk factor, the combination of relevant risk factors allowed for better stratification of immunotherapy in both responders and nonresponders.

Multiple clinical trials have either been conducted or in progress to evaluate the efficacy and safety of immunotherapy in treating AGC patients. The phase 2 single-arm KEYNOTE-059 study showed that among the previously treated AGC patients, the ORR of single-agent pembrolizumab was 11.6% overall [7]. The ORR was higher in the biomarker-positive population (15.5% in the PD-L1-positive population and 57.1% in those with MSI status) [7]. For the heavily pretreated patients, the phase 3 ATTRACTION-2 study demonstrated that there were survival benefits for patients treated with single-agent nivolumab compared with those treated with placebo [9]. However, the OS benefit of using nivolumab remained unaffected by PD-L1 expression levels in the ATTRACTION-2 study, which was possibly related to the method of detection [9]. The CheckMate-032 study made response comparisons between patients treated with nivolumab alone and those treated with nivolumab combined with ipilimumab in heavily pretreated patients. Combination therapy was associated with an increase in mPFS (2.2 months) and ORR (24%), reaching 40% in the PD-L1-positive population [24]. When compared to chemotherapy in the second-line setting, the single-agent pembrolizumab did not display significant survival benefit, with the median PFS of 1.5 months and median OS of 9.1 months in the KEYNOTE-061 study [17]. In comparison with chemotherapy, the response rate and survival of patients treated with single-agent pembrolizumab increased numerically, and the differences increased after patients were enriched by PD-L1 expression status. The benefits of immune combined chemotherapy have been reported by two phase III clinical trials in the first-line setting, but the conclusions are inconsistent. In KEYNOTE-062, pembrolizumab plus chemotherapy showed no superiority to chemotherapy (mOS = 12.5 vs. 11.1 months) in patients with a PD-L1 CPS of 1 or more [13]. Further analysis showed that a significantly higher ORR (57.1%) in the MSI-H population and a longer survival (17 months) in the population with CPS ≥10 were achieved adopting pembrolizumab alone. In CheckMate-649, the world's largest randomized phase III clinical study of GCs, nivolumab plus chemotherapy outperformed chemotherapy alone in OS (mOS 13.8 vs. 11.6 months) regardless of the PD-L1 expression. Further analysis showed that the OS differences became more and more obvious with the enrichment of patients by PD-L1 expression status [14]. Basically, the potential use of combination immunotherapy in biomarker-selected patients is supported by all of these studies. In fact, our study revealed that the ORR for combined immunotherapy was 24% and the mPFS was 5.8 months in the subgroup of patients who had previously received at least one line of systemic therapy. The subgroup of patients who received combination immunotherapy in the first-line setting conferred a more positive outcome, with the ORR of 48% and the mPFS of 7.1 months.

In our attempt to identify the subset of patients who responded to immunotherapy, we uncovered several highly relevant clinical and molecular biomarkers for immunotherapy response. PD-L1 expression and TMB are two independent biomarkers for immunotherapy response in most types of cancers [25–27]. Their roles in predicting the response of AGC patients was also confirmed by our results. Besides, lymphocyte count is related to the immunotherapy response because the activity of immune checkpoint inhibitors is dependent on functional T lymphocytes [28, 29]. In this study, the increased proportion of lymphocytes in the blood was reported for the first time as a strong and independent indicator for immunotherapy sensitivity in AGC patients. Such changes possibly reflect the cellular dynamics and functional adaptation of tumor microenvironment, thereby enhancing the antitumor effect of immune checkpoint inhibitors. In addition, the defects in DNA repair have been implicated in response to immunotherapy, as more neoantigens might be generated [30, 31]. We found that ATM, as one of the most frequently mutated genes in our AGC cohort, was associated with increased responses and survival outcomes. As all of these biomarkers are either established or highly relevant to immunotherapy responses, we evaluated the impact of their combinations in predicting the responses of immunotherapy. It was shown that integrating these biomarkers could help identify two distinct classes of patients with differential survival outcomes and responses to immunotherapy.

The current study has demonstrated the efficacy of combined immunotherapy in AGC patients both in the first-line and subsequent-line settings. Our findings have also underscored the value of an integrative approach with extensive clinical and molecular characterization to stratify patients with variable responses to immunotherapy. However, due to the limited sample size of the current study cohort, larger cohort studies are needed in the future to validate our findings.

## Figures and Tables

**Table 1:** Cohort characteristics.

Characteristics	No. (%) of total	No. (%) of NGS cohort
No. of patients	44	18
Age (years)		
Median (range)	60 (30–74)	62 (43–74)
Gender		
Male	29 (65.9%)	14 (77.8%)
Female	15 (34.1%)	4 (22.2%)
Smoking history		
Former smoker	20 (45.5%)	10 (55.6%)
Never smoker	24 (54.5%)	8 (44.4%)
Drinking history		
Former drinker	13 (29.5%)	7 (38.9%)
Never drinker	31 (70.5%)	11 (61.1%)
Family history		
Yes	7 (15.9%)	6 (33.3%)
No	37 (84.1%)	12 (66.7%)
No. of metastases		
1	18 (40.9%)	8 (44.4%)
>1	26 (59.1%)	10 (55.6%)
Liver metastasis		
Yes	25 (56.8%)	10 (55.6%)
No	19 (43.2%)	8 (44.4%)
Differentiation status		
Moderately differentiated	15 (34.1%)	5 (27.8%)
Poorly differentiated	20 (45.45%)	10 (55.6%)
NA	9 (20.45%)	3 (16.7%)
Lines of therapy		
One	23 (52.3%)	10 (55.6%)
Multiple	21 (47.7%)	8 (44.4%)
Therapy regimens		
I/O + antiangiogenic	24 (54.5%)	10 (55.6%)
I/O + chemotherapy	20 (45.5%)	8 (44.4%)

## Data Availability

The next-generation sequencing data used to support the results of this study are not yet available because we are still in the raw data upload stage.

## Supplementary Materials

Supplementary Materials Figure S1: efficacy of combination immunotherapy in AGC patients. Figure S2: clinical characteristics associated with immunotherapy responses. Figure S3: molecular characteristics associated with immunotherapy responses. Figure S4: association of PD-L1 expression status with immunotherapy.
