# Supplementary material for: Associations of Clinical and Molecular Characteristics with the Response to Immune Checkpoint Blockade in Advanced Gastric Cancers
Source: J Oncol. 2022 Mar 4;2022:2162229. doi: 10.1155/2022/2162229 (PMC8916884; doi:10.1155/2022/2162229)
Supplement: Supplementary Materials — Figure S1: efficacy of combination immunotherapy in AGC patients. Figure S2: clinical characteristics associated with immunotherapy responses. Figure S3: molecular characteristics associated with immunotherapy responses. Figure S4: association of PD-L1 expression status with immunotherapy. [file 2162229.f1.docx]

Supplemental Figure 1: Efficacy of combination immunotherapy in AGC patients

(A-B) Kaplan Meier plots of (A) progression-free survival and (B) overall survival for the study cohort. (C) Kaplan Meier plot of overall survival for patients treated with combination immunotherapy in the front-line or subsequent-line settings.

Supplemental Figure 2: Clinical characteristics associated with immunotherapy responses

(A-B) Kaplan Meier plots of (A) progression-free survival and (B) overall survival for patients with and without primary lesion resection. (C) Kaplan Meier plots of progression-free survival for tumors with different differentiation status. (D-E) Kaplan Meier plots of (D) progression-free survival and (E) overall survival for patients with high vs. low levels of lymphocyte count at baseline.

Supplemental Figure 3: Molecular characteristics associated with immunotherapy responses

(A-E) Kaplan Meier plots of progression-free survival for patients with and without (A) CCNE1 mutation, (B) CDH1 mutation, (C) APC mutation, (D) MYC mutation and (E) PI3K pathway gene mutations.

Supplemental Figure 4: Association of PD-L1 expression status with immunotherapy

(A) Kaplan Meier plot of progression-free survival for patients with different PD-L1 expression status. (B) Response rates for patients with different PD-L1 expression status.
